# Can We Prepare to Negate? Negation as a Reversal Operator

**DOI:** 10.5334/joc.119

**Published:** 2020-09-30

**Authors:** Carolin Dudschig, Barbara Kaup

**Affiliations:** 1 School of Psychology, Tübingen University, DE

**Keywords:** Word processing, Cognitive Control, Reading

## Abstract

Negation is a critical cognitive operator that is investigated across a wide range of psychological phenomena (e.g., language, eating control, emotion control, stereotype processing). A core function of negation is reversing input information. In the current study, we investigated whether this reversing process benefits from temporal preparation. In Experiment 1, participants were first presented with either the negator “not” or the affirmative counterpart “now”, and in a variable delay with the response indicating stimulus “left” or “right”. Participants had to respond according to phrase meaning (e.g., “now right” ➔ right response; “not right” ➔ left response). The results showed a persisting negation effect of 150 ms that did not reduce with preparatory time. In Experiment 2, we replicated this study using non-linguistic input information (i.e. crosses, tick-marks and arrows). Again, despite standard temporal preparation effects being present, the reversal process itself did not benefit from preparatory time. In summary, these experiments suggest that capacity demanding reversal processes are not eased if we know that a reversal process is coming up soon. This is particularly interesting, as in the current experiments a very basic binary negation paradigm was implemented. The implications of these results for models of negation processing are discussed.

## Introduction

Negation is typically an effortful and cognitively demanding process (e.g., Deutsch, Gawronski, & Strack, 2006; Deutsch, Kordts-Freudinger, Gawronski, & Strack, 2009; Dudschig & Kaup, 2018). To date, negation has been investigated across various research disciplines. For example, negation is of interest in philosophy, in linguistics, in animal research, and psychology. In psychology, research on the processing of negation has been carried out in various subfields, for instance in research dealing with language comprehension (e.g., Nieuwland & Kuperberg, 2008), in research investigating control processes related to action planning (e.g., Wegner, Ansfield, & Pilloff, 1998), emotion control (e.g., Herbert, Deutsch, Platte, & Pauli, 2012; Herbert, Deutsch, Sütterlin, Kübler & Pauli, 2011) and eating control (e.g., Adriaanse, van Oosten, de Ridder, de Wit, & Evers, 2011), as well as in research on rule-breaking (e.g., Pfister, Wirth, Schwarz, Steinhauser, & Kunde, 2016; Wirth, Pfister, Foerster, Huestegge, & Kunde, 2016), and stereotype modification (e.g., Gawronski, Deutsch, Mbirkou, Seibt, & Strack, 2008; Kawakami, Dovidio, Moll, Hermsen, & Russin, 2000). Recently, research into the mechanisms underlying the processing of negation has moved towards a more integrated approach, investigating negation as a central cognitive operation that falls-back on general cognitive mechanisms, such as inhibition or control (e.g., Beltrán, Muñetón-Ayala, & de Vega, 2018; Dudschig & Kaup, 2018; Dudschig & Kaup 2020; de Vega, Morera, León, Beltrán, Casado, & Martín-Loeches, 2016; Wirth, Kunde, & Pfister, 2019). Despite negation being such a central cognitive operator that is involved in various key cognitive domains, it is currently still widely discussed whether certain conditions help or hinder negation processing.

Given the diverse fields under which negation is investigated, we are currently far from a unifying explanation of the processes and mechanisms contributing to negation processing. One of the few agreements in the negation literature seems to be the fact that negation processing is typically difficult and demands increased cognitive resources. However, the source of this difficulty is still rather unclear. Intuitively, negation might be difficult to process because negated statements often leave open what the factual situation is. For example, when understanding a sentence such as “*The dress is not red*”, it remains unclear what colour the dress is (in the following non-binary negation). However, prolonged processing times for negated statements are also observed in situations where this is not the case. Such binary negation is present when the meaning of the negated statement equals the meaning of an affirmative statement (e.g., “*The door is not open*” => the door is closed), or when only two values of a certain attribute dimension are implemented such that a negative statement (e.g., “*The dots are not blue*”) allows inferring the factual property (e.g., “*The dots are red*”; for an overview, see Kaup & Dudschig, 2020). Taken together it seems that negation processing demands certain additional processing steps relative to affirmation, both for binary and non-binary negation. In the literature on negation processing, researchers have discussed several possibilities for such additional processing steps, namely processes related to inhibition or suppression (e.g., Giora, Fein, Aschkenazi & Alkabets-Zlozover, 2007; McDonald & Just, 1987), processes related to tagging information in the scope of the negation operator as false (Clark & Chase, 1972; Mayo, Schul, & Burnstein, 2004), as well as meaning-reversion processes particularly in the case of binary negation (e.g., Clark & Chase, 1974; Deutsch et al., 2006).

In the language processing literature, two factors have been investigated that presumably ease negation processing. First, it has been suggested that the pragmatically licenced use of negation makes negation integration accessible and easy, and second, it has been suggested that additional time facilitates negation processing. With regard to pragmatic licensing, it has been argued that negation is such a common phenomenon in everyday language that there must be situations in which negation processing takes place rather spontaneously without demanding additional processing time or resources. Accordingly, several studies have aimed at investigating negation in language situations in which negation is used in a pragmatically licensed manner (e.g., “*With the proper equipment, scuba-diving isn’t very dangerous*”) and indeed observed that negation integration took place instantly during comprehension (Nieuwland & Kuperberg, 2008). Nieuwland (2016) extended these findings and showed that such initial integration only takes place in situations where negation occurs in sentences that are highly constraining and therefore evolve in a highly predictable manner. However, such pragmatic licencing of negation is not present and also not possible in all situations of negation use. As mentioned above, negation as a cognitive operator is also implemented in other domains such as eating control, emotion control or as an instruction to reverse certain input information and is not even always used in a linguistic format. For example, when investigating the comprehension of traffic signs involving symbols indicating “no right turn” (Walker, Nicolay, & Stearns, 1965; see also Giora, Heruti, Metuki & Fein, 2009) or in situations where negation is used as a maker to indicate to reverse certain information (e.g., stereotype processing, mental control). In these cases, there seems no instance of licencing the use of negation. Indeed, negation has been shown to be extremely difficult to process under all these circumstances.

In addition to the benefits from pragmatic licencing of negation use, it has been suggested that unlicensed linguistic negation integration benefits from additional processing time. In the studies by Kaup et al. (2006), additional time after encountering the negated proposition (e.g. “*The door is not closed*”) allowed comprehenders to integrate the negation into the meaning representation. Here, following a delay of 1500 ms, an image depicting the factual situation (e.g., an open door) was processed faster than an image depicting the non-factual situation (e.g., a closed door); see also Hasson & Glucksberg, 2006). However, in these studies, the additional time was given after both the negator and the to-be-negated proposition was presented. Thus, these studies do not allow conclusions regarding whether additional time to deal with the negator itself facilitates subsequent core processes of tagging, suppressing or reversing of information. In contrast, these studies show that if there is additional processing time after encountering the full to-be-negated information, comprehenders are indeed able to integrate un-licenced negations to a level that the final meaning represents the factual states of affairs (see also Dudschig & Kaup, 2018). In this context, an electrophysiological study by Dudschig, Mackenzie, Kaup and Leuthold (2018) is of particular relevance. In this study, it has been shown that negation does not benefit from additional time if provided prior to the information in the negator’s scope (e.g., “*It is not true that ladybirds are stripy*”). Critically however, this study did not involve binary negations, or in other words, did not implement attribute dimensions that allowed the inference of the factual state of affairs from a negated statement (if a ladybird is not stripy, we don’t know what it is instead). Therefore, it remains to be answered whether those aspects of negation processing that are presumably involved in the processing of binary negation can be eased by additional time. In the current study, we focus on processes related to meaning reversal. We consider it likely that in the processing of binary negation (e.g., “not left” ➔ right keypress; “not right” ➔ left keypress) preparatory time allows preparing the reversal process. Here, we aim to investigate such negation-related reversal processes by analysing whether preparatory time helps with these processes.

In cognitive psychology, temporal preparation is a well-studied phenomenon. When executing standard choice or simple stimulus-response tasks, it is well known that two core factors influence response time: event preparation and temporal preparation (Jennings & Van der Molen, 2005). Temporal preparation is typically investigated by means of presenting a warning signal that allows participants to prepare for the upcoming response without specifying response parameters (e.g., Niemi & Näätänen, 1981). Also, other mental operators, such as task switching highly benefit from temporal preparation (e.g., Meiran & Daichman, 2005). In the current study, we aim to investigate whether temporal preparation will facilitate the upcoming reversal processes as a key component of processing binary negation. Therefore, in our current experiment we use the phrases “not left”, “now left”, “not right” and “now right”. Participants are asked to respond according to phrase meaning (Dudschig & Kaup, 2018). Importantly, in this study, we vary the temporal distance between the presentation of the negation information (i.e. “not” vs. “now”) and the response triggering stimulus (i.e. “left” or “right”) across five fixed intervals, giving the participants more or less time to prepare for the upcoming reversal process. In a previous study we showed that a similar paradigm – without manipulating temporal distance – resulted in an increase of processing time by approximately 150 ms in the negation compared to the non-negation condition, and even at the end of the experimental session, the negation effect persisted (Dudschig & Kaup, 2018). Electrophysiological data supported the claim that such a type of negation demands a cognitive reversal process, as initially incorrect motor activation was found over the motor cortex ipsilateral to the response side. Only later this activation was reversed to result in activation of the correct motor cortex. In the current experiment, we expect in line with the temporal preparation literature reaction times to be fastest in the longest stimulus onset asynchrony (SOA) condition (e.g., Niemi & Näätänen, 1981; Trillenberg, Verleger, Wascher, Wauschkuhn, & Wessel, 2000). Second, if reversal processes in this setup can be prepared with additional time, we expect a reduction of the negation effect with increasing SOA. In contrast, if reversal processes are not facilitated by additional preparation time, we expect the negation effect not to interact with the SOA.

In our view, the results of the current study will also be informative with respect to a classic question within the negation processing literature. According to the two-step model of negation processing, comprehenders grasp the meaning of a negative sentence by mentally simulating two states of affairs. In a first step, they simulate the non-factual state of affairs (i.e., a stripy ladybird for “this ladybird is not stripy”). In a second step, they turn their attention away from this simulation and instead focus on simulating the factual state of affairs (i.e. a dotted ladybird, or a ladybird without a specific pattern in case the comprehender lacks the respective knowledge). Importantly, the first processing step (simulating the non-factual state of affairs) is an integral part of negation processing, both for binary and non-binary negation (cf. Kaup, Yaxley, Madden, Zwaan, & Lüdtke, 2007; Kaup, Zwaan, & Lütke, 2007). In contrast, according to the fusion model of negation processing, comprehenders only engage in the first processing step for negative sentences with non-binary negation where the comprehender does not have enough knowledge to simulate the factual state of affairs (e.g. “The dress was not red” – here it remains unclear what colour the dress was). For binary negation (e.g. “The door is not open”), however, the comprehender presumably fuses the negation operator with the respective attribute and arrives at the factual state of affairs (= the door is closed) within one processing step (e.g., Horn, 1989; Lyons, 1995; Mayo, Schul, & Burnstein, 2004). Concerning the question investigated in the current experiment, namely whether negation-related reversal processes are facilitated by additional time between processing the negation operator (i.e., “not”) and processing the spatial term (i.e., “left” vs. “right”), the two processing models of negation make different predictions. According to the two-step model of negation processing, comprehenders always need to mentally simulate the negated state of affairs in order grasp the meaning of the negated phrase. For “not left” they need to simulate “left” and for “not right” they need to simulate “right” in a first processing step. As the content of the simulation depends on the spatial term, additional time between negation operator and spatial term should not facilitate negation processing. In contrast, according to the fusion model, the binary negation in phrases such as “not left” and “not right” in the context of an experiment with only two spatial terms allows for the possibility that comprehenders directly fuse “not right” into “left” and “not left” into “right”. From the perspective of this processing model, it therefore seems well conceivable that additional time between processing the negation operator and processing the spatial term might allow participants to prepare the reversal process and therefore facilitate negation processing. Thus, in our view, finding evidence for facilitation by additional time in the current paradigm would be more in line with the fusion model than with the two-step model of negation processing.

## Experiment 1

In the first experiment we used standard linguistic negation whereby the word “not” or “now” indicated whether participants either had to respond to the meaning of the following spatial term “left” or “right” or had to reverse their response. The phrases “not” and “now” were temporally separated from the spatial terms in order to investigate the influence of preparation on the subsequent reversal process.

### Methods

#### Participants

20 participants were tested, *M*_age_ = 25.25, *SD*_age_ = 4.12, range = 19 to 36, 16 female, 19 right handed. Sample sizes were determined using the partial eta square form the previous study investigating negation in a similar experimental setup (Dudschig & Kaup, 2018), resulting in a sample size of 8 participants in order to replicate the negation effect. As we were interested in investigating an influence of an additional factor (SOA) on the negation effect, we increased sample size to 20. We had planned to remove the data of individual participants from the subsequent analysis if their overall error rate was greater than a predefined error rate of 20%. No data had to be removed. Participants were paid 8€/h or received course credits. All participants provided signed informed consent prior to the experiment.

#### Stimuli and Procedure

The experimental procedure was programmed in Matlab using the Psychtoolbox 3.0.15 (Kleiner, Brainard, Pelli, Ingling, Murray, & Broussard, 2007) and presented on a Linux computer running Ubuntu 18.04 displayed on a CRT. All stimuli were displayed in black (RGB 0,0,0) on a white (RGB 255,255,255) background. Each trial started with a fixation cross presented for 750 ms (ca. 0.5 cm × 0.5 cm). This was followed by the presentation of the German word for “not” (=nicht) or “now” (=jetzt) for 300 ms (ca. 1 cm × 0.5 cm). Subsequently, there followed a random SOA of 100 ms, 300 ms, 600 ms, 1000 ms or 1500 ms with a blank screen until the response triggering German directional word (“left” (=links) or “right” (=rechts)), was presented. The word took up approximately 1.3 × 0.5 cm. The word stayed on the screen until response or for 2000 ms if no response occurred. It followed a 1000 ms interval until the fixation cross was presented again. If participants did not respond within 2000 ms, the feedback “too slow” was displayed for 1000 ms. If participants responded anticipatory and thereby faster than 150 ms (see Dudschig & Kaup, 2018), the feedback “too fast” was displayed. In the case of an error, the feedback “error” was displayed. The experiment consisted of six experimental blocks with 100 trials each, resulting in 60 trials in each experimental condition. The experiment started with a practice block consisting of 20 trials.

#### Design

The experimental design was a 2(polarity: affirmative vs. negated) * 5(SOA: 100, 300, 600, 1000 vs. 1500 ms) repeated measures design. The dependent variables were reaction times (RT) and error rates.

### Results

**Figure 1:**
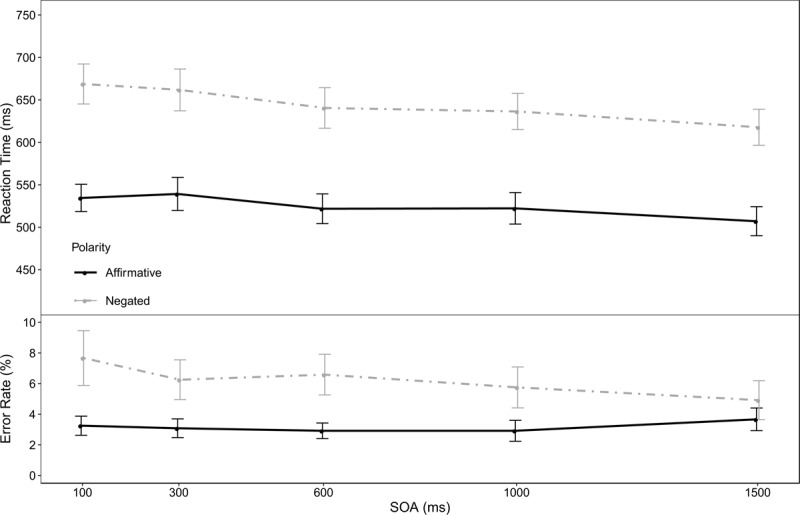
Reaction time (top) and error rate (bottom) of Experiment 1 as a function of polarity (negation vs. affirmation) and SOA. The error-bars represent ±1 *SEM*.

The overall error rate was 4.83%. Individual trials classified as too fast (<150 ms) or too slow (>1500 ms) were removed from the subsequent reaction time analysis, resulting in 0.55% percentage of trials removed as outliers. The analysis of the RTs showed a main effect of negation *F*(1,19) = 170.86, *p* < .001, η^2^_p_ = .90 (*M*_aff_ = 525 ms, *M*_neg_ = 645 ms). There was also a main effect of SOA, *F*(4,76) = 11.41, *p* < .001, η^2^_p_ = .38 (*M*_100_ = 602 ms, *M*_300_ = 601 ms, *M*_600_ = 581 ms, *M*_1000_ = 579 ms, *M*_1500_ = 562 ms). Importantly for the current question under investigation, there was no interaction between negation and SOA, *F*(4,76) = 1.24, *p* = .30, η^2^_p_ = .06, suggesting that additional time to prepare for the upcoming reversal process did not facilitate it. The error rates mirrored the main findings: There was a main effect of negation, *F*(1,19) = 10.92, *p* < .001, η^2^_p_ = .36 (*M*_aff_ = 3.17%, *M*_neg_ = 6.23%) and no interaction between negation and SOA, *F*(4,76) = 1.57, *p* =.21, η^2^_p_ = .08. The SOA however did not influence the error rates, *F*(4,76) = 0.66, *p* = .55, η^2^_p_ = .03. Mean reaction times and error rates in the ten different conditions are displayed in Figure 1.^1^

### Discussion

The current experiment shows that additional time to prepare for an upcoming negation does not benefit the negation process. Both the RTs and error rates were affected by negation and did not significantly benefit from increased preparatory time regarding the negation process. This is rather surprising, especially given that in the current experimental setup only two directional words (left vs. right) were used. Therefore preparation should, in principle, be possible following the processing of the negation operator, at least according to the fusion model of negation processing. The current results therefore underscore the wide held assumption that negation processing is inherently difficult and fits well with the view that negation processing involves two processing steps, even in case the negation is binary.

According to the two-step model of negation processing (e.g. Kaup et al., 2007), the first processing step of simulating the non-factual state of affairs reflects pragmatic aspects of language processing. The contexts in which negation is used during everyday communication are rather limited. Negation is typically used to communicate exceptions from rules or expectancies or to correct a previously made assumption (for an overview, see Moeschler, 1992). When encountering a negation out of the blue, comprehenders can therefore be assumed to infer the respective context, accommodating the rule, the expectancy or the previous statement. Thus, when encountering a sentence such as “The ladybird is not stripy” comprehenders are assumed to “infer” that there is reason to assume that the ladybird is stripy or that someone must have made such a statement. This accommodation of the missing context is reflected in the first simulation step during which a stripy ladybird is being conceptualized. In a pragmatically felicitous context, this accommodation is either unnecessary or particularly easy, and negation processing is thus facilitated. As pragmatics is part of the linguistic system, it seems conceivable that these kinds of accommodation processes are limited to negation use in language. In the next experiment, we therefore investigated whether negation processing can indeed be facilitated by additional time in case of non-linguistic negation. Maybe here, the first simulation step is not necessary, and the fusion model is the better description of the processing of negation.

## Experiment 2

The results of Experiment 1 show that negation integration processes do not seem to benefit from preparatory time. Experiment 2 was designed to replicate Experiment 1, but this time using a non-linguistic type of negation and non-linguistic directional symbol. This was done to investigate whether the findings from Experiment 1 are due to specifics in the linguistic system. Thus, Experiment 2 was identical to Experiment 1 with the only difference being that instead of words, pictorial negation symbols and arrows as directional cues were used (see also Dudschig & Kaup, in revision).

### Methods

#### Participants

20 participants (*M*_age_ = 26.5, *SD*_age_ = 5.05, range = 19 to 37, 13 female, 16 right handed) took part in this experiment. As in Experiment 1, it was planned to remove the data of individual participants from the subsequent analysis if their overall error rate was greater than a predefined error rate of 20%. In this experiment, this resulted in the removal of the data of one participant. Participants were paid 8€/h or received course credits. All participants signed informed consent.

#### Stimuli and Procedure

The procedure was identical to Experiment 1, but this time instead of the linguistic words a pictorial stimulus type was used. These stimuli were all unicode symbols (check mark (✔) with decimal code: ✔ ballot x (✗) with decimal code ✗ leftwards arrow (←) with decimal code: ← rightwards arrow (→) with decimal code: &#8594).

#### Design

The experimental design was a 2(polarity: affirmative vs. negated) * 5(SOA: 100, 300, 600, 1000 vs. 1500 ms) repeated measures design. The dependent variables were reaction times (RT) and error rates.

### Results

**Figure 2:**
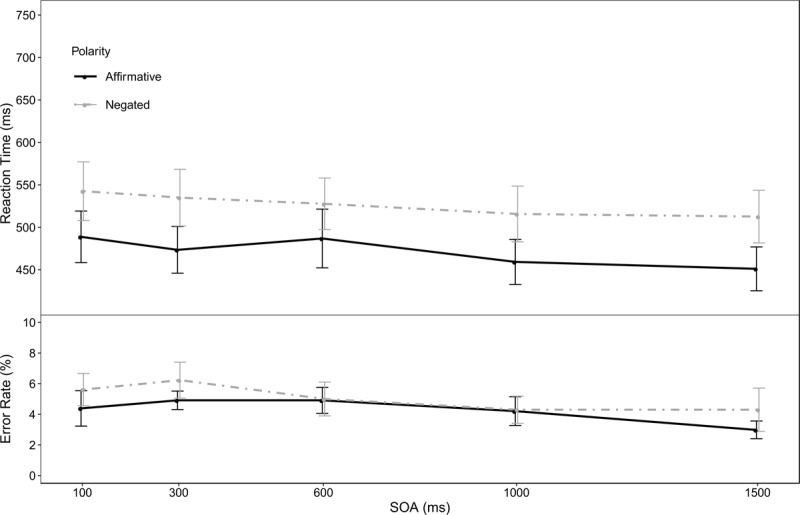
Reaction time (top) and error rate (bottom) of Experiment 2 as a function of polarity (negation vs. affirmation) and SOA. The error-bars represent ±1 *SEM*.

The overall error rate was 6.51%. Individual trials classified as too fast (<150 ms) or too slow (>1500 ms) were removed from the subsequent reaction time analysis, which resulted in the removal of 1.02% of the trials. There was a main effect of negation, *F*(1,18) = 45.37, *p* < .001, η^2^_p_ = .72 (*M*_aff_ = 472 ms, *M*_neg_ = 527 ms). There was also a main effect of SOA, *F*(4,72) = 8.99, *p* < .001, η^2^_p_ = .33 (*M*_100_ = 516 ms, *M*_300_ = 504, ms, *M*_600_ = 507 ms, *M*_1000_ = 487 ms, *M*_1500_ = 482 ms). As in Experiment 1, there was no interaction between the negation effect and the SOA, *F*(4,72) = 0.94, *p* = .42, η^2^_p_ = .05. The error rates showed no significant effects (main effect negation: *F*(1,18) = 1.19, *p* = .29, η^2^_p_ = .06; main effect SOA: *F*(4,72) = 2.17, *p* = .09, η^2^_p_ = .11; interaction: *F*(4,72) = 0.47, *p* = .70 η^2^_p_ = .03). Mean reaction times and error rates in the ten different conditions of Experiment 2 are displayed in Figure 2,^2^.

### Discussion

Experiment 2 was planned as a conceptual replication of Experiment 1 with the use of pictorial instead of linguistic input. The core results replicated the findings from Experiment 1, suggesting that negation processing is similar for linguistic and non-linguistic negation, in particular concerning the view that negation processing involves conceptualizing what it is that is being negated during a first processing step. Nevertheless, there were some surprising differences, especially with regard to the negation effect. First, the negation effects seemed much smaller in Experiment 2 than in Experiment 1. Second, the error rates were not affected by negation in this experiment, in which a non-linguistic input format was used. In order to determine whether these differences were significant, a post-hoc analysis was performed directly comparing the two experiments. Therefore, we ran an ANOVA with the three factors: polarity, SOA and experiment. The analysis of the RTs showed a main effect of experiment, *F*(1,37) = 5.91, *p* = .02, η^2^_p_ = .14, due to RTs in average being faster in Experiment 2 (*M*_pictorial_ = 499 ms) compared to Experiment 1 (*M*_linguistic_ = 585 ms). Additionally, there was the to-be-expected main effect of polarity, *F*(1,37) = 205.52, *p* < .001, η^2^_p_ = .85, due to negation trials (*M*_neg_ = 587 ms) being slower than affirmative trials (*M*_aff_ = 499 ms). Also the main effect of SOA was significant, *F*(4,148) = 18.78, *p* < .001, η^2^_p_ = .34, due to a decrease in RTs with increasing SOA. Most interestingly, the interaction between Experiment and polarity was significant, *F*(1,37) = 28.03, *p* < .001, η^2^_p_ = .43, indicating that there was a smaller negation effect in Experiment 2 (*M*_neg_ – *M*_aff_ = 55 ms) than in Experiment 1 (*M*_neg_ – *M*_aff_ = 120 ms). No other interaction was significant. In the analysis with error rates, only the effect of polarity was significant, *F*(1,37) = 10.84, *p* < .001, η^2^_p_ = .23, with smaller error rates in the affirmative than in the negated condition. There was no significant interaction with the factor Experiment.

## General Discussion

Despite negating information being a core cognitive function – also linguistically present in every language (Horn, 1989) – it is still debated why negating is so difficult and how this process could be facilitated. In the negation processing literature, there is clear evidence that pragmatic licencing eases negation integration (Nieuwland, 2016; Nieuwland & Kuperberg, 2008) and additionally that time is a crucial factor for negation integration (e.g., Kaup, Lüdtke & Zwaan, 2006). Interestingly, up to date it remains unclear how this factor of time actually helps with the specific process of negating. The current experiments were designed to investigate the potential benefit of temporal preparation for negation processing. Two experiments – using both linguistic and pictorial negation – showed that additional preparatory time does not benefit the core process of reversing. Participants were presented with the negator “not” and subsequently had between 100 ms and 1500 ms to prepare for the upcoming reversing process. However, this additional time to prepare did not ease the actual impact of negation. Specifically, across all five SOA conditions there persisted a negation effect of approximately 150 ms. This is specifically surprising given the fact that in the current study an experimental setup was implemented that demanded a binary reversing process given the input information. What could be the reasons making negation processing such a difficult process?

Negation has been shown to be hard to comprehend, difficult to integrate into action plans and to demand specific licencing processes in numerous studies. Nevertheless, it has been demonstrated that even subliminal presentation of a negator implemented in negator-noun pairs (e.g., pick baby vs. not baby) can be processed to a level that it correctly modifies subsequent tendencies towards the choice of the correct noun (e.g., baby vs. other word (e.g. yard)) (Armstrong & Dienes, 2013). Importantly, this study focused on showing that even a subliminally presented negator can influence subsequent choices. However, this study did not demand a full integration of the negator and the noun, and the results therefore do not necessarily indicate that participants engaged in reversal processes. Interestingly, the current study shows that in situations where negation is used to reverse the given input information, there seems hardly any possibility of preparing for this reversal process. As this holds both for linguistic and pictorial input information, it can be concluded that negation under these circumstances does not benefit from preparatory time. For linguistic input, one might argue that specifics in the linguistic input might hinder such a preparatory process. However, for visual input in the form of arrows, one might assume that during a preparatory time, an internal preparation for the upcoming reversal process might take place, which was not the case.

As argued in the introduction, we consider this result to fit well with the view that negation processing involves two processing steps (Kaup et al., 2007), even in the case of binary negation. When grasping the meaning of “not left”, comprehenders must first grasp the meaning of “left” and only in a second step integrate the negation and arrive at the factual situation (right). The first step depends on the spatial term used in the current trial and processing can therefore not be sped up by additional time between encountering the negation operator and the spatial term. The fact that additional time did not lead to processing ease for both linguistic and non-linguistic negation suggests that the first processing step is not only required for linguistic negation processing, but also for non-linguistic negation processing. This seems to speak against the idea that pragmatic aspects are at the root of the first processing step. Alternatively, pictorial negation might be similarly dependent on context as linguistic negation. After all, pictorial negation is probably also mainly used in situations in which the state of affairs that is being negated constitutes at least a possibility. For instance, it would probably be surprising to encounter a crossed out left-pointing arrow as a road sign in a situation in which there is no left turn road.

There is, however, another interesting aspect supported by the current two experiments, that actually does point towards a difference in the way the linguistic and pictorial information is processed. Negation processing often triggers ironic effects of negation (Wegner, Ansfield & Pilloff, 1998): Ironic action effects occur when a comprehender executes exactly the response or behaviour that should explicitly *not* be executed. For example, Wegner, Ansfield and Pilloff report that during putting a golf ball or swinging a pendulum, participants more likely performed the to-be-avoided type of action (e.g., don’t overshoot -> overshooting) than in conditions in which they were not explicitly instructed to avoid this exact type of behaviour. Such effects are not only observed in the domain of action planning, but also for mental control (e.g., “*Don’t think about the pink elephant*”, Wegner & Erber, 1992) or eating control (Adriaanse et al., 2011). One could argue that in the current experiment such ironic effects of negation were observed in the error data: Especially in Experiment 1 using linguistic negation, participants exhibited an increased error rate in the negation condition – showing that they are more likely to execute the to-be-negated response (e.g., “not left” -> participants press the left response key). Interestingly, this pattern did not occur when using pictorial input format in Experiment 2, suggesting that ironic action effects are specifically triggered by linguistic input in such an experimental setup. Therefore, indeed specificities in the linguistic system seem to underlie the way negation is processed. One explanatory attempt would be to argue that ironic effects reflect the first processing step and that this is more pronounced for linguistic than non-linguistic negation because of pragmatic aspects of language. However, as argued above, if non-linguistic negation processing does not involve the first processing step then we would have expected to see facilitatory effects in the longer SOA conditions for non-linguistic negation, which is not what we observed. Therefore, it seems unlikely that differences with respect to pragmatic aspects explain the differences between the two input formats. A further difference between the two input formats related to the size of the negation effect. Pictorial negation led to a smaller negation effect than linguistic negation. One possible interpretation is that linguistic negation is highly associated with prohibition during language acquisition and might therefore carry a highly unpleasant connotation. Maybe some part of the negation effect in linguistic processing indeed reflects processing difficulties due to this negative connotation (for an overview see Kaup & Dudschig, in press). Our studies do not allow to draw definite conclusions with respect to the discussed explanations of format differences. Further studies are clearly needed to better understand when and how negation processing in different input domains differ, and which types of input domains are more likely to trigger erroneous behaviour.

There is a final major question that needs to be addressed with regard to the current study and findings: In how far can the results be explained from an action-control perspective? In both experiments participants operate a spatial stimulus-response task (see Miles & Proctor, 2012). It could now be argued that in the affirmative condition participants can formulate basic stimulus-response bindings, where the stimulus word “left” (vs. “right”) is bound to the left vs. right response – a congruent stimulus-response code mapping. In the negated condition such bindings are resulting in a conflicting representation, as participants need to link the word “left” (“right”) to a right (vs. left) response. In such a view, the incongruency between the stimulus and response code in the negated condition can explain the main effect of negation. Also, in the Miles and Proctor study (Experiment 3, incompatible condition) participants were instructed to press the opposite direction of what is implied by a word, location or arrow stimulus. In line with the results of the current study, the spatial compatibility effects were stronger for words than for arrows under such conditions (cf. Vu & Proctor, 2004). Critically, in the current study we don’t have a general instruction to follow that instructs to reverse the stimulus code to the opposite, instead in each trial we have the negation or affirmation cue that indicates whether participants need to follow the standard stimulus-response mapping or whether they need to reverse the mapping. From an action-control perspective it might be highly unlikely that participants are able to prepare for such cued reversal process. Indeed, our study shows that such a reversal process is extremely difficult.

In the present paradigm, there was a relatively tight coupling between the linguistic codes and the expected behaviour. Therefore, the findings of the present study possible do not generalize to all types of language processing where the coupling is often more diffuse. However, it should be noted that also in everyday language there sometimes exist exactly such a tight coupling where language is used to instruct or inhibit certain behaviour, especially with the use of negation (e.g. “*Don’t run across the street*”). In any case, the results of the present study indicate that reversal processes that are typically involved in the processing of certain types of negations cannot be effectively prepared. Thus, the question outlined in the introduction whether adding more time in between the negation and the information in its scope would facilitate the reversal process can be answered with no.

In summary, to date it has been shown that pragmatic licensing of negation – in a manner that allows prediction of the upcoming information – allows instant negation integration to occur. The current study shows that temporally preparing for an upcoming reversal process does not ease the reversal process itself. Therefore, it seems that negation that calls for reversing the input information is a highly demanding process for the cognitive system. Such findings are in line with the two-step model of negation processing according to which grasping the meaning of a negative sentence requires grasping the meaning of what is being negated as a first processing step. These results are also consistent with previous claims regarding ironic effects of negation, indicating that negation probably should be avoided for instructions even if there is sufficient preparatory time. Interestingly, especially in the linguistic experiment, the error rates remained increased in the negation condition, directly supporting that idea that ironic effects of negation occur across all preparatory intervals to a similar level.

## Data Accessibility Statement

Data will be made available upon publication on the Potsdam Mind Research Repository (http://openscience.uni-leipzig.de/index.php/mr2).

## Footnotes

In addition, the Bayes factor was determined (Wetzels, van Ravenzwaaij, & Wagenmakers, 2015). For the RTs, the test of the model with only the main effects against the model containing the interaction between polarity and SOA resulted in a Bayes factor of approx. 10, which can be interpreted as strong evidence against the interaction. For the analysis of error rates a Bayes factor of approx. 7 resulted, providing moderate evidence against the interaction.

In addition, the Bayes factor was determined. For the RTs the interaction between polarity and SOA resulted in a Bayes factor of approx. 10, which can be interpreted as strong evidence against the interaction. For the analysis of error rates a Bayes factor of approx. 15 resulted, providing strong evidence against the interaction.
